# Proximal radius fracture morphology following axial force impact: a biomechanical evaluation of fracture patterns

**DOI:** 10.1186/s12891-019-2529-9

**Published:** 2019-04-06

**Authors:** Lucca Lacheta, Sebastian Siebenlist, Maximillian Lauber, Lukas Willinger, Nicole Fischer, Andreas B. Imhoff, Andreas Lenich

**Affiliations:** 1 Department of Orthopaedic Sports Medicine, Technical University of Munich, Ismaninger Strasse 22, 81675 Munich, Germany; 2 Department of Orthopaedic and Trauma Surgery, Helios Hospital Munich West, Steinerweg 5, 81241 Munich, Germany; 3 Arthrex Department of Research & Development, Erwin-Hielscher-Strasse 9, 81249 Munich, Germany

**Keywords:** Radial head fracture, Biomechanics, Fracture patterns

## Abstract

**Background:**

The most common location for articular fractures of the radial head is often reported to be the anterior lateral aspect of the radial head with the arm in neutral position. However, these findings mainly base on clinical observations rather than precise biomechanical measurements. The purpose of this study was to evaluate the formation of proximal radius fractures, the association between axial forces and fracture morphology, energy to failure and bone stiffness in a biomechanical in-vitro setup.

**Methods:**

18 fresh-frozen cadaveric radii performed axial load compression with 10 mm/min loading until bone failure. Energy to failure and bone stiffness were recorded. Proximal radial head fracture morphology and affection of the anterolateral quadrant were optically analyzed.

**Results:**

All radii survived a compression load of 500 N. The mean compressive forces that lead to failure were 2,56 kN (range 1,30 – 7,32). The mean stiffness was 3,5 kN/mm (range 2,0 – 4,9). 11 radial neck fractures and 7 radial neck and radial head multifragment fractures were documented. The anterolateral quadrant was involved in 78% of tested radii.

**Conclusion:**

The anterolateral quadrant of the radial head (in neutral position of the forearm) is confirmed to be the most common location for articular radial head fractures in a biomechanical setting. In case of a fall on the outstretched arm radial neck fractures should be securely ruled out due to prior occurrence to radial neck and head fractures.

## Background

Fractures of the radial head are the most common fractures of the elbow [1]. The mostly reported injury mechanism for radial head fractures is a fall on the outstretched forearm with axial compression of the radial head against the capitulum [2]. Previous observations suggested that the most common location for articular radial head fractures is the anterolateral quadrant with the forearm in neutral position [3, 4]. Quantitative computer tomography (CT) measurements confirmed the anterolateral quadrant of the radial head to be most affected [5].

Several hypotheses exist explaining why the anterolateral quadrant is mainly involved. While some authors report of the lowest bone density in the anterolateral quadrant [6], others postulate the orientation of forearm, radial head and wrist during load transmission at time of impact to be crucial [7].

However, these are clinical findings and, to the best of our knowledge there are no biomechanical investigations evaluating the relation of isolated axial load forces and fracture morphology of the radial head. As Amis et al. published in 1995 the indirect biomechanical loading of elbow specimen showed characteristic fracture lines with predisposed radial head fractures at the anterolateral rim [8]. However, their biomechanical testing did not allow to answer the question if directly applied forces in 0° flexion (fall on the outstretched arm) will cause the longitudinal fractures seen clinically.

Therefore, the purpose of the present study was to confirm the aforementioned clinical observations and to describe the fracture patterns following isolated, direct axial impact to the radial head. Additionally, the energy to failure and bone stiffness in a biomechanical in-vitro setup should be evaluated in order to better understand proximal radius fracture formations. The hypothesis was that clinical and radiological observations that radial head fractures mostly occur anterolaterally can be confirmed in biomechanical setup with isolated axial force application.

## Methods

### Specimens

A total of 18 fresh-frozen cadaveric radii were used in this study. Institutional review board approval (study number 188/18 s) was obtained. All specimens were male with a mean age of 55,2 (range 19–64) and mean body mass index of 23,7 kg/m^2^ (range 14,7 – 34,9). Soft tissues were completely removed from the bone of the proximal radius. The radii were shortened with a consistent length of 16 cm from the radial head. All radial head joint surface diameters were 26 mm measured with a radius gauge prior to testing.

### Biomechanical testing

**Fig. 1:**
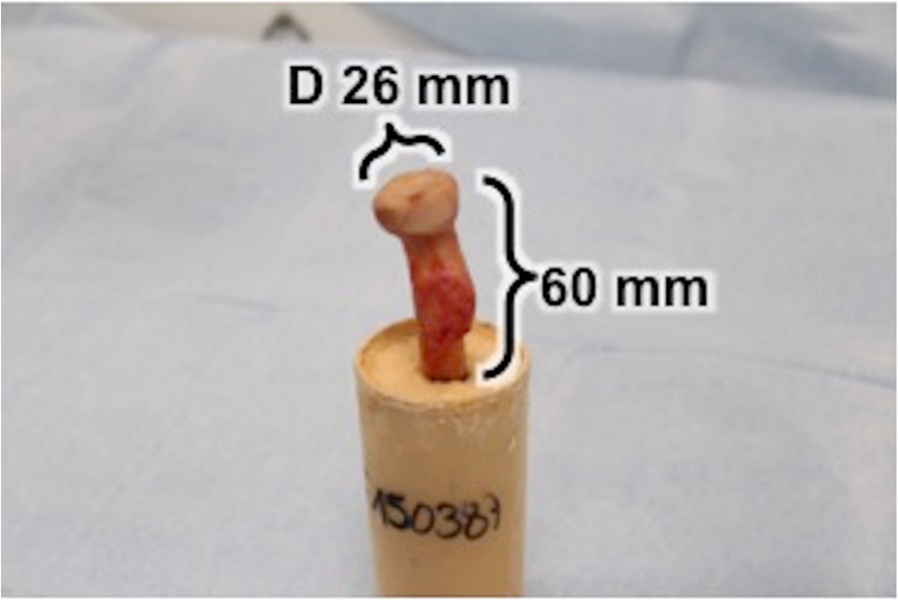
Shortened proximal radii with a consistent length of 6 cm and completely removed soft tissue fixed perpendicular to the ground. Radial head joint surface diameters were 26 mm measured with a radius gauge prior to testing

**Fig. 2:**
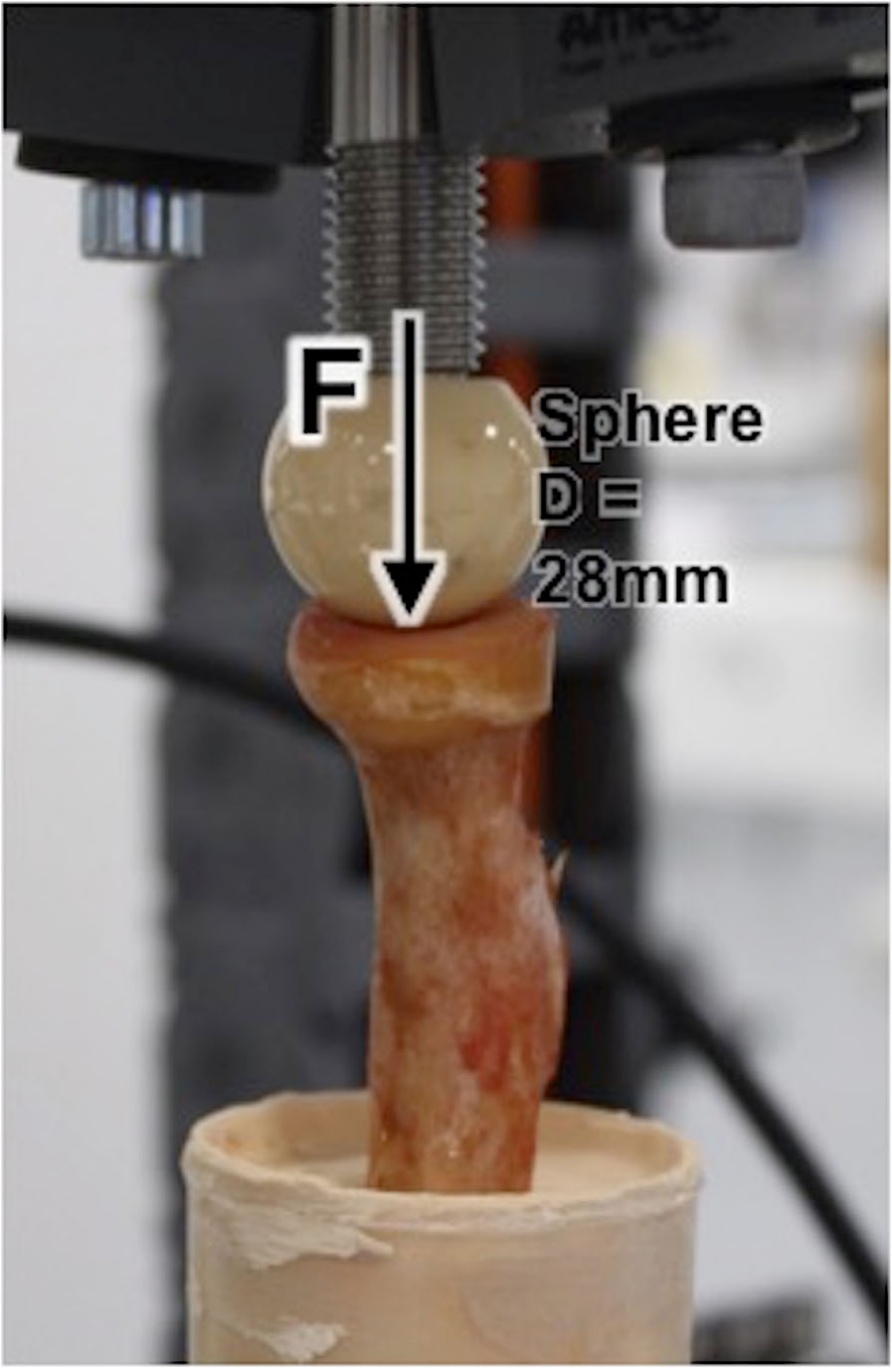
Specimen-cylinder mounted on material testing machine Instron E 10000. Force transmission was applied within inner radius via spherical head harvested from hip prosthesis axial to the radial shaft

Specimens were securely fixed with super hard stone type 4 (Hera Moldasynt, compressive strength 54 MPa) in custom-made cylinders. 60 mm of the proximal radial shaft from the radial head were detached perpendicularly to the ground (Fig. 1). Each specimen-cylinder was mounted on a material testing machine Instron E 10000 (Instron Cor., Darmstadt, Germany) (Fig. 2).

Force transmission was applied within inner radius via a spherical head harvested from hip prosthesis (Fa. Zimmer) with a diameter of 28 mm which corresponds to the radiocapitellar ratio as described by Sandman et al. [9] (Fig. 2). Load was applied in the mechanical axis of the radial shaft to simulate the in-vivo injury mechanism (fall on the outstrechted arm). The perpendicular positioning of the radial shaft was ensured by the use of bubble level during embedding. A static axial compression loading with 10 mm/min was applied until bone failure (= fracture). Bone failure was defined as sudden loss of measured force > 50% and visible deformation of the radial head.

### Data collection and statistical analysis

Loading (kN) and compression set (mm) were recorded. The bone stiffness of each specimen was calculated from the compression set under the applied loading. A minimum of 5 views were photographed of each testing to determine bone deformation and fracture morphology.

All data were presented as mean and standard deviation (SD) for normal distributed variables. Level of significance was set at *p* < 0,05. The sample size of 18 human cadaver was determined based on a previous study by Gordon et al. with similar outcome parameters suggesting a required sample size of 10 to 16 specimens. To generate enough power a sample of 18 specimens was chosen [10]. Analysis was performed with the SPSS software, version 22 (SPSS Inc., Chicago IL) statistical package, for Windows.

## Results

All 18 radii completed the axial force compression setting until bone failure. All specimens survived at least an axial compression force of 500 N. The mean compressive forces that lead to failure were 2,56 kN (range 1,30 – 7,32).

Based on a load of 500 N (for the highest value all radii were intact) a calculation of stiffness was performed. At this load mean stiffness was 3,5 kN/mm (range 2,0 – 4,9).

**Fig. 3:**
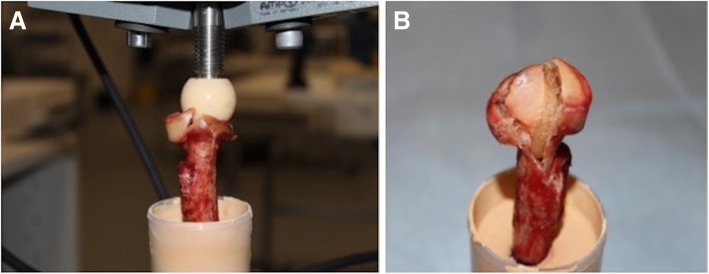
Proximal radius after axial load was applied (**a**) resulting in a radial neck and head fracture (**b**)

**Fig. 4:**
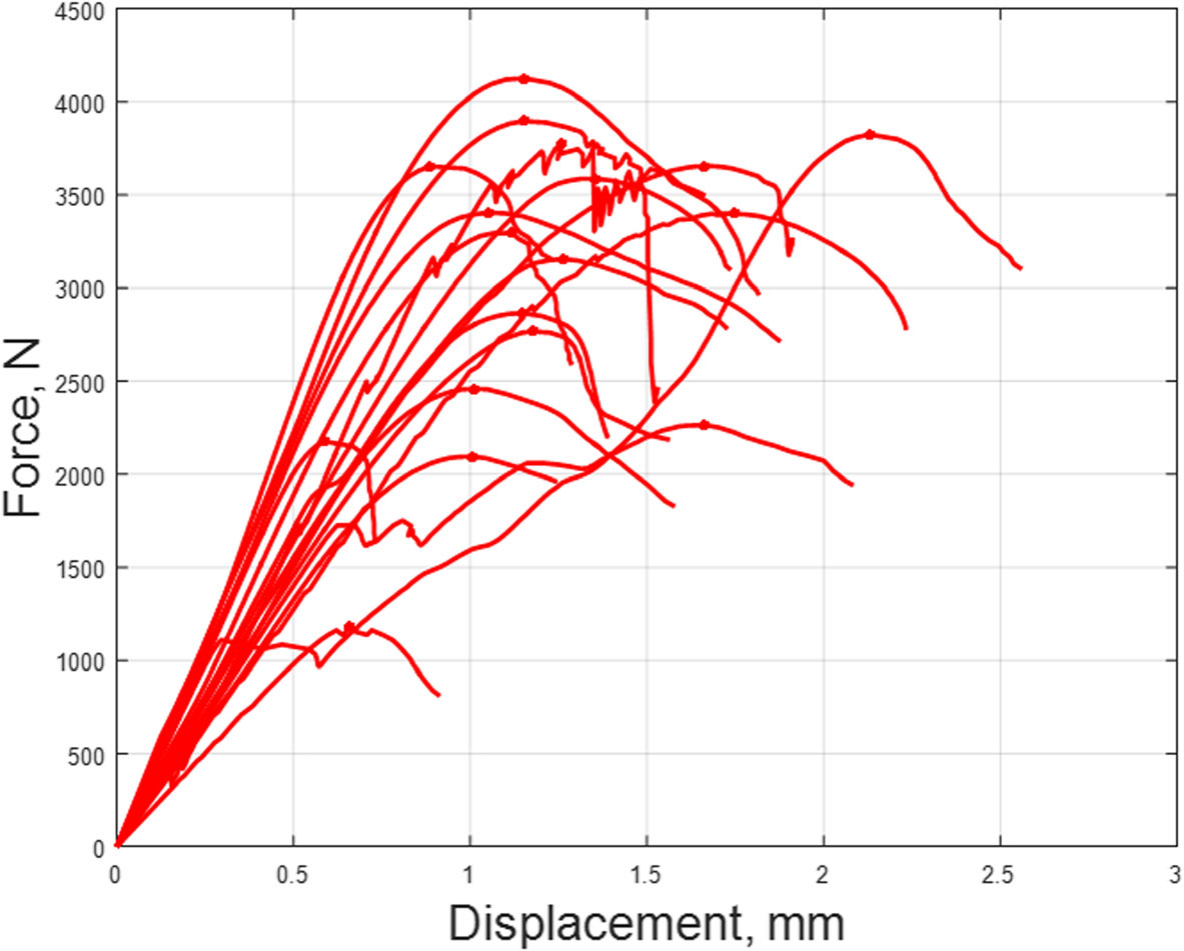
Mean deformation (mm) of the proximal radius under the applied load (N)

11 radial neck fractures and 7 radial neck and head multifragment fractures were documented. First radial neck fractures (Mason Type III) were observed before radial head multifragment fractures occurred. The anterolateral quadrant was involved in 78% (14 out of 18) of fractures. Exemplary fracture morphology under the applied load and mean deformation of the proximal radii are shown in Figs. 3 and 4.

No correlation between age and stiffness (R = 0.5), or age and failure load (R = 0.4) were observed. Furthermore, no correlation between and fracture pattern was detected.

## Discussion

This biomechanical in-vitro study confirmed the clinical observations that the anterolateral quadrant is the most common location for articular radial head fractures. The main finding of this study is that radial neck fractures occur first (Mason Type III, AO A3, B3, C3) before radial head multifragment fractures rise secondly.

Previous observations already suggested that the most common location of radial head fractures is the anterolateral quadrant of the radial head in neutral position [11]. However, these are clinical findings, quantitative measurements of proximal radius fractures are rare. While the injury mechanism for proximal radius fractures with fall on the outstretched forearm seems to be clear, the exact formation of proximal radius fractures especially the predisposition for fragments at the anterolateral quadrant and the role of geometry properties, bone quality and elbow stability with translational and rotational forces is still unclear.

Imaging studies are showing that the anterolateral quadrant has the lowest bone density which might explain the preferred fracture location anterolaterally [6, 11]. Whereas anatomical investigations demonstrated that the bone strength at the anterolateral aspect of the radial head is comparable to other quadrants with no mean difference in indentation modulus and local yield strength across all 4 quadrants as shown by Gordon et al. and in CT absorptiometry measurements by Eckstein et al. [10, 12]. According to our results, van Leeuwen et al. [5] although confirmed the anterolateral quadrant as the most common location in radial head fractures in 92% (22 of 24) in quantitative CT-scan measurements of fracture lines. Van Leeuwen et al. underline the importance of the radial head in elbow stability and hypothesized that the position of the forearm during trauma mechanism may influence fracture location but limited that a lack of characterization of radial head fractures exists to improve the understanding of treatment and outcomes of these fractures. Rosenblatt et al. [13] support the hypothesis that translational and rotational forces lead to specific fracture patterns at the radial head preferred anterolaterally and describe the role of the anterolateral radial head as a buttress against posterior dislocation of the elbow during encountering forces during injury e.g. fall on an outstretched arm. As shown by Amis et al. in a biomechanical investigation using impact tests in cadaver specimens with soft tissue, radial head fractures in an outstretched arm lead to a rim fracture in over 80%. This group called for a biomechanical investigation with an axial, direct force to the radial head to better understand the longitudinal fracture lines seen clinically. However, this was not possible in their biomechanical setup [8]. Therefore, this study tried to answer the question of proximal radius fracture patterns with isolated axial force impact (without rotational forces) and to compare the findings to clinical observations.

Our study weakens the hypothesis that rotational loads are crucial in developing radial head fractures at the anterolateral quadrant of the radial head as seen clinically. The anterolateral quadrant was even most affected (78%) in isolated axial force application without rotational or translational forces suggesting that geometry and bone quality may play the major role in fracture formation and morphology. We hypothesize that the increased density and bone volume as shown by Haverstock et al. may act as a protective factor to the posteromedial quadrant vice versa predisposes the anterolateral quadrant for frequent fractures [6]. Of course, rotation and translation forces as well as valgus movements may contribute to an overload anterolaterally but may not have the major effect on fracture morphology.

The study should be interpreted in respect to several limitations. First, this study is not considering ligamentous and muscular stabilizing properties of the elbow during proximal radius fracture injury mechanism. Second, rotation and translation loads were not considered. Third, the radius-capitulum-ratio in this study is only an approximation to in-vivo conditions and not displaying anatomical circumstances exactly. Fourth, radial head fractures are most common in men and women between 30 and 40 years. The tested radii were older and exclusively male, which should be considered when interpreting the study findings.

## Conclusions

The anterolateral quadrant of the radial head (in neutral position of the forearm) is confirmed to be the most common location for articular radial head fractures in a biomechanical setting. In case of a fall on the outstretched arm radial neck fractures should be securely ruled out due to prior occurrence to radial neck and head fractures.

## Abbreviations

CT: Computer tomography
SD: Standard deviation
